# A Smart Recommender Based on Hybrid Learning Methods for Personal Well-Being Services

**DOI:** 10.3390/s19020431

**Published:** 2019-01-21

**Authors:** Rayan M. Nouh, Hyun-Ho Lee, Won-Jin Lee, Jae-Dong Lee

**Affiliations:** Department of Computer Science, Dankook University, 152 Jukjeon-ro Campus, Suji-gu, Yongin-si 16890, Gyeonggi-do, Korea; rayannouh@gmail.com (R.M.N.); leehh4016@naver.com (H.-H.L.); god7300@dankook.ac.kr (W.-J.L.)

**Keywords:** machine learning, hybrid recommender system, dynamic well-being services

## Abstract

The main focus of the paper is to propose a smart recommender system based on the methods of hybrid learning for personal well-being services, called a smart recommender system of hybrid learning (SRHL). The essential health factor is considered to be personal lifestyle, with the help of a critical examination of various disciplines. Integrating the recommender system effectively contributes to the prevention of disease, and it also leads to a reduction in treatment cost, which contributes to an improvement in the quality of life. At the same time, there exist various challenges within the recommender system, mainly cold start and scalability. To effectively address the inefficiencies, we propose combined hybrid methods in regard to machine learning. The primary aim of this learning method is to integrate the most effective and efficient learning algorithms to examine how combined hybrid filtering can help to improve the cold star problem efficiently in the provision of personalized well-being in regard to health food service. These methods include: (1) switching among content-based and collaborative filtering; (2) identifying the user context with the integration of dynamic filtering; and (3) learning the profiles with the help of processing and screening of reflecting feedback loops. The experimental results were evaluated by using three absolute error measures, providing comparable results with other studies relative to machine learning domains. The effects of using the hybrid learning method are gathered with the help of the experimental results. Our experiments also show that the hybrid method improves accuracy by 14.61% of the average error predicted in the recommender systems in comparison to the collaborative methods, which mainly focus on the computation of similar entities.

## 1. Introduction

The term “well-being” is mainly considered as a positive outcome, and refers to the process of evaluating people in terms of being satisfied with their life. Regarding the World Happiness Report, it includes aspects of life on the social and personal levels that comprise health and economy as critical variables for personal well-being [1,2]. Diseases are prevented with the help of physical activities, personal behavior, nutrition, and lifestyle. There is also a reduction in treatment cost, leading to gradual improvements in life expectancy, energy level, and good healthy feelings [3,4,5,6]. In this context, various studies have targeted the effective development of personalized systems for healthcare. Requirements of healthcare systems are found to be increased due to the increased adoption of technologies for an active and healthy lifestyle. An integrated system for healthcare consists of effective capabilities that lead to stability regarding individuals’ awareness of health [7,8,9]. With regard to the advancement of personalized technologies, there has been increased research focusing on the success of digital recommender systems concerned with health. The existing systems for healthcare, which mainly include digital health devices such as Apple Watch, Google Fit, and Samsung Health, effectively operate for individual health and fitness tracking. These devices help by providing efficient and recommended features that work on the effective analysis of the human body, including footsteps, heart rate, and hours of sleep [10,11,12,13,14,15]. These systems have the additional feature of focusing on the tracking of fitness. However, concerning the development of eating habits, these measures are found to be ineffective to sustain a healthy lifestyle. In order for users to develop efficient and healthy habits with these systems, they must generate proper guidance for nutrition based on recommendations of services for well-being [16]. For effective understanding of the functions of the core system, it is observed that the recommender systems can contribute to the improvement of quality related to the information overload involved in semantic technology.

Primarily, recommender user ratings effectively respond to the functions as vectors [17]. In this context, the concept of ontology contributes to the matching of similarities with the help of various filtering techniques. In a similar context, collaborative filtering methods are also implemented by researchers involved in the development of recommender systems [18]. There are various large-scale studies in the literature that focus on hybrid methods using different techniques of filtering, specific health conditions, and healthcare monitoring systems [19,20,21]. As there are various prototypes developed by practitioners and researchers involving well-being factors in the recommender systems, the benefits of learning with the help of the recommendation process are effectively recognized and promoted [22]. However, the systems cannot be recognized as sufficient to change the impoverished lifestyle of users. To change users’ unhealthy habits, there is a huge requirement that the recommendations be actionable [16].

The existing research mainly aims at monitoring health and fitness with the help of user devices without considering the contextual information and nutritional components [22]. To determine the contextual information, various learning methods have been proposed. The existing recommender system method integrates the core functions with the help of collaborative filtering that involves few limitations related to the recommendations for the cold start. Regarding the two types of cold start problems, the new user problem is considered to be more difficult [23,24,25,26,27,28]. Accordingly, some studies have proposed hybrid methods, which mainly combine content-based filtering and collaborative filtering to enhance the limitations with the help of algorithms [29].

**Figure 1:**
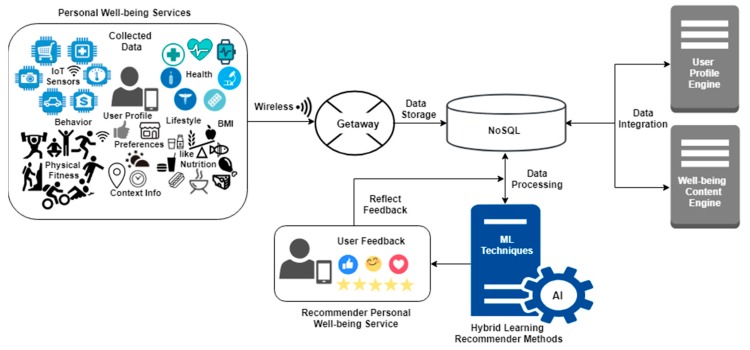
Well-being recommender services.

With regard to the rising amount of information and data, various challenges in the filtering methods have been seen to arise, including leanness and scalability [30]. The latest trends in machine learning employ various layers of processing, which helps in learning from complex contextual features. It has also been observed to be pervasive, as the effective ingredients of the recommender system provide accurate services for users. According to the experts, it has been found that machine learning has the power to determine the factors involved in health and lead to improvements in quality of life with the help of lifestyle health services [31,32,33]. Figure 1 depicts the large set of data that was collected with the help of various types of sensors to address the inefficiency of the technologies and the effective approaches that have been developed by researchers.

Information in the context of well-being is collected and transformed by connecting personal identities with mobile devices by the use of getaway of wireless nature. NoSQL [30] is used to store data, which helps in integrating contextual information in two engines, profile engine and well-being content engine. Each technique focuses on different facets by the use of machine learning techniques. Hybrid learning methods give the content of a personalized recommender and are based on feedback of a reflecting nature. There are challenges in recommendation, which can be represented as the estimation of the response of a new user, choosing specific algorithms, learning mechanism, and the evolution of performance [31]. Well-being content supports recording routine activities and provides the recommender service usage. The handling of data requirements is done with communication of data sources in many aspects. There is integration of data related to the context of users and engines of well-being services.

The processing of data is based on supervised machine learning algorithms that learn from reflective functions, which in turn helps in utility prediction and recommended well-being services for every user. The research work helps in capturing the focus of researchers, because the problem of cold start arises when a user profile does not exist in the system, and there is no available previous rating [32]. In order to tackle these challenges, in this paper a new learning method based on machine learning is proposed for an advanced recommender, which relies on hybrid learning methods to improve upon current recommender systems using collaborative filtering for better personal well-being services. 

The main motivation of this work focuses on solving the cold star and scalability issues in a collaborative filtering method. To effectively address the inefficiencies, this paper presents a hybrid learning methods, SRHL which is a smart recommender system of personal well-being in regard to health food service. SRHL has been designed to examine how combined filtering approach improve crucial issues of cold star problem using a profile learning loop concept, and what kind of user feedback reflected to improves the recommender accuracy and enhance the efficient performance based on machine learning techniques. With regard to the research, the most essential and suitable contextual features used to develop the effective recommendations mainly include time, activity, location, and the composite information related to monetary costs, ingredients, health and nutritional value, availability, and the effects of combining the ingredients. In relation to meeting the requirements, the methods of hybrid learning are integrated with various algorithms, including: (1) switching among content-based and collaborative filtering to resolve the cold start problem for new users; (2) identifying user context within the dynamic filtering; and (3) integrating the profile learner methods to reflect user feedback.

In this regard, the expected and suitable contributions are as follows: (i) improving the new user problem of the cold start; (ii) optimizing the precision of the hybrid recommender system in the domain related to the services of health food for personal well-being; (iii) enhancing the user experience with the help of relevant and effective suggestions depending on the user’s identified context; and (iv) showing evidence of the concept, which involves a learning loop that contributes to continual advancement in the performance of the personalized recommender. All of these contributions lead to effective enhancement of the pre-existing methods of recommender learning and address the extraordinary challenges found in the prediction of well-being services.

The major outcomes of this paper mainly focus on the classes of existing recommender systems. The evaluations of the parameters adopted by efficient supervised learning contribute to the analysis of validity related to the efficiency of methods involved in the prediction of learning methods. The performance is measured using multiple metrics to evaluate the accuracy of recommendations, based on three performance errors: mean squared error (MSE), mean absolute percentage error (MAPE), and mean absolute error (MAE) [34].

The remaining parts of the paper are mainly structured in sections that include a review of the literature focused on the background for the analysis of the present situation, explained in Section 2. Section 3 consists of explanations regarding the proposed methods of hybrid learning, including the system architecture, functional algorithms, and effective parameters. The results of the validation process are explained in Section 4, and the conclusion along with future work are discussed in Section 5.

## 2. Related Work

### 2.1. Recommender System

A recommender system (RS) provides correct information needed by a particular user so that the user can choose the best in specific fields. This puts RS on the list of successful software tools [10]. Netflix, YouTube, Amazon, and other platforms have been viewed as successful in terms of providing recommendations to users; the objective is to give users content that is more personalized [16]. The individualized content minimizes the information load for users and provides refined content that users want. In addition, it also enables fast reactions to situations, decision-making, and understanding of users’ needs [19]. The recommender system’s classification has various types of filtering, such as hybridization and collaborative filtering, filtering on the basis of content, and collaborative filtering. The collaborative filtering method is used by most existing recommendation systems [25]. This filtering has problems at the initialization of the system because a new user has no rating or previous data in the rating table. In order to overcome this situation, the system gives suggestions considering the interests of users similar to those of the new user. The method in which filtering is performed on the basis of content views the user’s objections and shows recommendations as per the history of the user’s likes. Based on previous research, the shortcoming of the RS is overcome by the use of a combination of two methods [16,17,18,19,20,21,22,23,24,25,26].

As discussed above, the method used in most recommender systems shows the most relevant information per the user, ignoring other information related to the user such as weather, time, and location (for example, information related to watching movies in the most suitable theater or having dinner at a healthy restaurant) [23]. The dynamic information of user contacts has been used in previous research related to recommender systems. These studies show that to provide recommendations, it is important to incorporate the information related to the user in the RS. A model of contextual filtering also has some limitations related to its application in a single dimension, as in the case of a user’s neighbor. The studies show that the demand for services for personal well-being by the RS is a crucial part of the services of personal health care [35,36]. This can provide services that reduce the efforts of persons doing a specific task, which helps to improve individuals’ lifestyles by giving personalized medicines and digital health, and this also helps to reduce the expenditure for the treatment. Using techniques of learning, the RS can increase the level of predicting and evaluating the components at multiple levels with the help of dynamic information in order to provide a good user experience [37].

### 2.2. Machine Learning

As per artificial intelligence (AI), the tasks of the recommender system can be processed on the basis of the user profile, which represents the user’s behavior [38]. This is the reason for the popularity of machine learning (ML) in learning the techniques of the personalized system and its application in the future of certain domains [39]. ML tries to compile traits and probabilities on the basis of the factors of the risk result from the lifestyle. Techniques of ML are capable of creating a model that provides information about personal emotions to accumulate the interest of users. In addition, ML techniques are also used to take content from the entire pool of content and provide relevant information and improve the performance of the recommendations [40,41,42,43].

A significant amount of research has been carried out on ML techniques, which has drawn attention to the recommender system. The system presents the advantages of helping users operate online. Online users are encouraged by the content alternatives they are provided. Given the use of many algorithms in the literature, four primary techniques of learning methods are included [44]. The methods can be categorized as guided, unguided, and reinforcement methods. In the guided method, all the information has labels. Training information is fed in by a formula to anticipate the result, but this is not the case in unguided training. In unguided training, the emphasis is on seeking patterns in the data that are not clear. In a semi guided algorithm, incomplete sets of training are used to make predictions [45]. In the reinforced methods, the agent is rewarded based on its performance in learning to act. The results in this method are predicted by another thinking source [46]. Present studies show that techniques of machine learning and methods of filtering are mostly used in recommender systems. There are also hybrid methods, which can be used to improve the recommendations with the help of machine learning techniques [47].

### 2.3. Well-Being Food Services

Recommended well-being services have become an essential platform in the field of lifestyle and health [48]. Nutrition and these systems have been fixed in a service that is capable of enhancing the uses of overburdened information, which is reducing over time. There have been fewer studies conducted in the field of human nutrition because food is an essential part of health and morals [49]. People in food professions have an ethical duty, along with their professional duty, to provide food. This particular field of study has gotten tied up in different subjects and food morals regarding its important characteristics. Recommender systems in the lifestyle context are used to assist in diagnosing for health advising [50]. AI is a field with an increasing scope for further studies in the context of machine learning, exposing humans to new possibilities in studies affecting the changing recommender systems in services related to food and well-being. New methods in this field have unbelievably complicated components, but they can be made simpler by saying that they are about human health [51]. The main component in this paper is the hybridization of the machine learning method. It is essential to keep the rate of anticipation high in the context of user behavior while forming these learning methods to enhance system efficiency.

## 3. Proposed SRHL System

**Figure 2:**
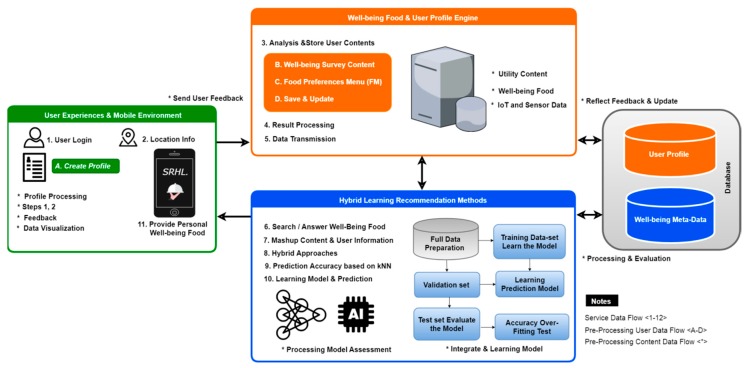
Service model.

The critical part of providing a smart recommender hybrid system of learning (SRHL) consists of user profiles and well-being healthy food content in a hybrid approach. The concepts of technology development, the system outline, the structure, and service use cases for the model designed for well-being content for free care fusion in the context of the above configuration are described in the service flow. Figure 2 shows the concept of the SRHL service model and specifies the components of each service. In step 1, users answer a simple survey to determine their dietary restrictions and nutritional expectations. This information will be saved in the user’s profile history to filter healthy food items and create an initial set of recommendations. In step 2, users make use of an adaptive visual interface to express their fine-grained food preferences through simple comparisons of food items.

The learned preferences are used to further re-rank the recommendations presented to them. In the rest of this service model, depicting the process of the proposed methods, the first model is data acquisition, which involves collecting information as mentioned in steps 1 and 2. The second model service data flows from <1–12>, which can be transmitted and stored as preprocessed user data flow specified in <A–D> locally in well-being and the user profile engine. The third model is the hybrid learning methods required for preprocessing content data flow mentioned in <*> using ML to train and test the dataset to evaluate the learning production model to improve the accuracy over the hybrid filtering test and preserve the results based on k-nearest neighbors for the classification algorithm, and the results are presented and discussed in the next section. The recommendations will provide more appropriate suggestions according to the user’s targeted preferences based on a reflected user feedback loop. Well-being content is combined to define a care fusion type. Well-being Health food service paradigm and metadata contents are determined in order to construct a food preference menu. It also receives and analyzes users’ demographic, health, and personal information to create profile-authoring technology through the proposed service model.

### 3.1. SRHL System Architecture

**Figure 3:**
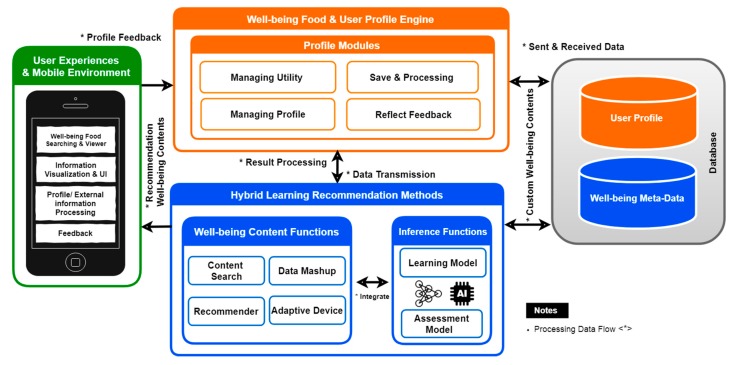
System architecture.

As discussed in the previous section on the SRHL, the proposed system architecture is shown in Figure 3, and consists of three main models, user experiences in the mobile environment to create a user profile, then data processing into profile modules that can manage utility, save and leading profile and reflect user feedback. The hybrid learning method consists of well-being content and inference functions for learning and assessment of the production model to better match the needs. Hybrid learning methods customize well-being content to recommended learning methods due to its process predication accuracy using feedback loop classification. An overview primarily comprises well-being complex content-authoring technology with the architecture design based on the available IEEE standard options [10]. The Software Requirements Specification (SRS) provides the basic guidelines for the proposed system. The architecture follows standards that include functional and nonfunctional requirements. The learning methods in inference functions are based on the k-nearest neighbor algorithm to process preferences in user rating scores and ranking of food content [45]. In the next step, the recommendation engine provides users with optimal recommendations based on the functions of the learning methods according to their feedback loop and preferences. Figure 3 shows the specific technologies required for each component.

### 3.2. SRHL Learning Methods

**Figure 4:**
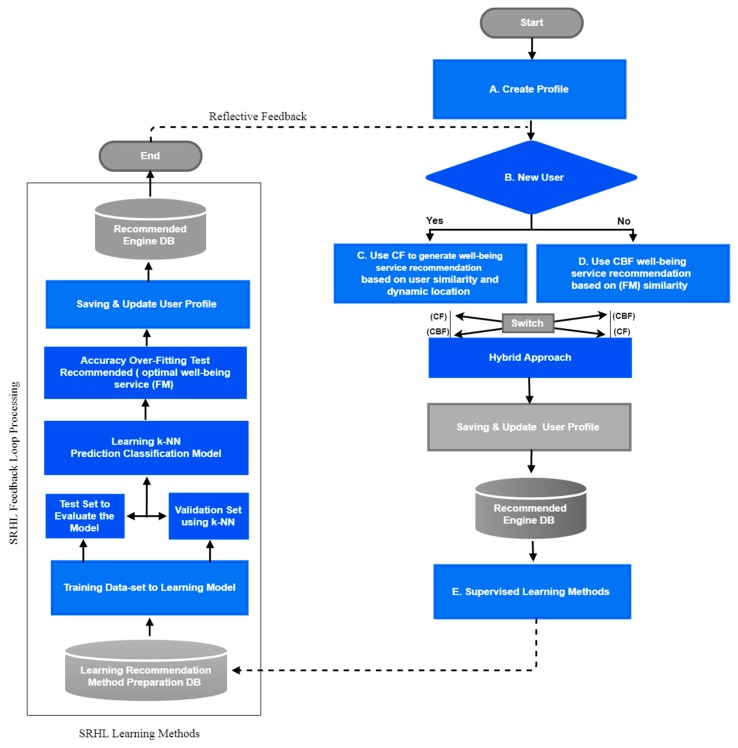
Methods.

The core idea behind SRHL processing is to compute the similarity index between users and well-being content by analyzing user profiles and learning methods from feedback loop to mash-up using the new optimized recommender in a mobile environment. The learning methods assessment flowchart, shown in Figure 4, consists of multiple functions that will be described below. Table 1 contains the specific proposed method parameters, which assign different value definitions to explain the processing of the algorithm.

The algorithm shown in Figure 4 is a step-by-step method for a hybrid learning technique, the process of the algorithm to be understood. In this method, a user has to create a profile, in which the given function elaborates the anticipation step after giving the user ID as input and shows the substitute for food. The substitute food menu (FM) is suggested based on the FM list that has already been rated by the user. In the next step, the learning techniques create a set of information in the context of well-being using the straining model by taking inputs from the observations. The next step in the process is to filter the data and select the items that have been liked or rated by the user. The same process is repeated to generate a similar set of information. The results are not independent any more. The method is called hybrid learning, so the results from both iterations are combined. Common points in the attributes of FM and collaborative filtering (CF) are the basis of content-based filtering (CBF). This calculates the similar natures between FM and U. The learning method of the hybrid process keeps switching from one straining to another in order to resolve the cold start problem for a new user. As explained in the flowchart, guided learning of machines is applied in the paper. This also elaborates the hidden points, various patterns, and connections from a set of information generated during training in the categorization of multiple classes with the help of k-nearest neighbor to generate the operations that can measure the similarity in their characteristics, and this will give feedback in the form of a loop that will help to enhance the user experience.

#### 3.2.1. Create a Profile

**Figure 5:**
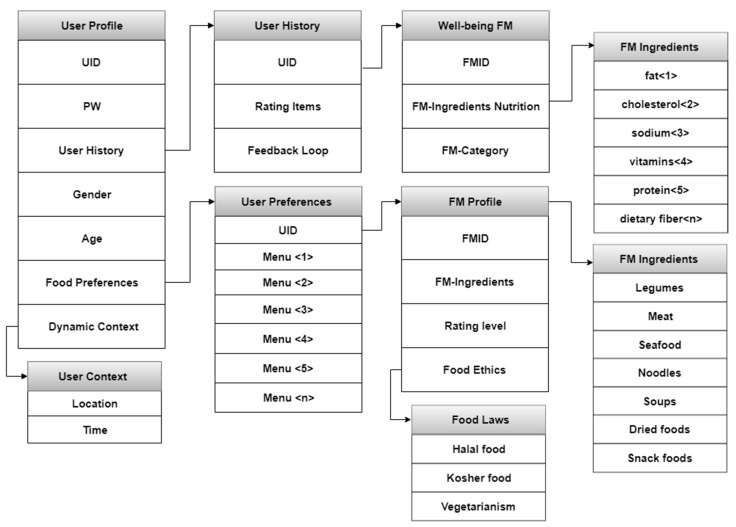
Profile structure.

The module of the profile comprises utility management, vectors of the profile, saving, and reflection of feedback. Each module consists of a user history and well-being food content, which reflects the user preferences. This basic step helps to create recommendations of a personalized nature and gathering of preferences related to learning methods. Figure 5 depicts the profile structure and processing, which helps in gathering and saving user data, and then analyzing them. This helps in the prediction of user patterns, which is related to previous actions.

The profile creation depicts and creates the main function of RS, configuration of the user profile related to personalized fusion services of well-being. The user can assess the relevant features and symbols, and an analysis provides a selection of recommendations for well-being food content. This allows the user to agree to the requirements of the profile information, which allows automation of processes and the correct content is delivered. The feature and preference information is used to generate the user’s profile information. With a huge amount of data, it becomes vague and leads to unclear meaning if precise definitions are not used. There is a loss of significance of classified data even the user is classified, and there is the extraction of characteristics. The data of unfounded classification and feature extraction are not useful, and user satisfaction cannot be confirmed regarding the content recommendations. There are six categories of profile configuration, and each helps in the extraction of the profile with full and accurate optimization. These subdivisions of items help in fast and accurate processing of the profile.

#### 3.2.2. New User

This function addresses the problem of cold start, which occurs when a new user is registered [27]. No previous rating of this user is found in the rating table in the system to improve the cold start, which is a big issue in RS. It pertains to the sparsity of information about users and items adapting to a user. The system needs to know the user history, such as which content the user most liked in the past. However, when a new user joins the system, nothing is known about the user. Thus, it is not possible to make RS with content-based filtering that is built on community preferences such as ratings, the core functions mentioned in Figure 1 and Figure 4.

#### 3.2.3. Collaborative Filtering

Collaborative filtering (CF) uses automatic prediction filtering about user preferences by collecting more feedback regarding interests. With ratings and taste information from many users, we used this approach to get more similarity between users and generate recommendations based on user similarity and dynamic location information. This result is conceptually quite simple: given a user-U, find a set of other users-U who have rated some of the same well-being food service content as U. It recommends a menu that is popular in U_a_ and is based on the recommendation approach, meaning that the algorithm does not need to know anything about the lists themselves, except their identity food IDs. The idea is to recommend foods that are popular among users who like the same menu as specific users. In practice, this is done by first building a user-menu rating matrix like the one shown in Table 2.

The ratings in the recommendation engine make it easy to find the set of users who share ratings with any given user. They also explain the algorithm for calculating the similarity between two users, given the user FM matrix. The similarity value can thus range from 0 to −1, where 1 represents perfect results. For example, if two users, U_3_ and U_5_, do not share any ratings, their similarity score will be 0, while −1 shows that users have opposite opinions about the FM, such as U_5_ and U_6_, and will have a similarity score approaching 0. However, users who rate the same list equally, such as U_1_ and U_4_, will get a score of 1.

The pseudocodes below explain the process of obtaining similarities between users. It has a significant influence on the final recommendations compared to less similar users. To recommend an FM preference list to U_2_, first calculate the similarity scores for the set of users who have rated some of the same FM as U_2_. In this case, users U_1_, U_3_, U_4_, and U_5_ would all get a similarity score of 1, which matters the most when calculating the top FM list. However, as U_3_ has not rated any FM other than the one shared with U_2_, we need to consider only U_1_, U_4_, and U_5_. FM_2_ is popular with these users, while U_2_ has not rated it yet. Therefore, FM_2_ is the generated list that will ultimately be recommended. The similarity value can thus range from 0 to −1, where 1 represents perfect results. The Algorithm 1 below explains the process of obtaining similarities between users [25]. It has a significant influence on the final recommendations compared to less similar users.


**Algorithm 1: Predicting similarities between users**

#Return a Euclidean distance similarity score for U_1_ and U_2_define sim_score (prefmatrix, $U_{1}$, $U_{2}$):$\mathrm{sim}\left(U_{1},U_{2}\right)=\frac{U_{1}\cdot U_{2}}{\left|\left|U_{1}\right|\right|\left|\left|U_{2}\right|\right|}$# assume U has no rating and feedbacksi = Falsefor menu in prefmatrix [$U_{1}$, $U_{2}$]:if menu in prefmatrix [FM]:si = Truebreak# if they have no rating in feedback, return 0if not si:return 0
**# add the squares of all differences**
sum_of_squares = $\mathrm{sim}\left(U_{1},U_{2}\right)=\frac{\sum \left(U_{1},U_{2}\right)}{{\sigma U}_{1}\times {\sigma U}_{2}}$sum ([pow (prefmatrix [$U_{1}$] [FM] − prefmatrix [$U_{2}$] [FM], 2)For menu in prefmatrix [$U_{1}$]
If menu in prefmatrix [,$U_{2}$]])then ignore score sim <1# return the similarity as a value between 0 and 1return 1/(1 + sum_of_squares)


The previous methods explain how to calculate similarities between two users using Euclidean distance. It is shown that both users have ratings in the FM list preference matrix. In this algorithm, the prediction uses user rating preferences for the FM list by adding all distance similarity scores between two users. To calculate the predicted ratings for each FM, ignore the value if less than 1 for similar users and add the squares of all differences for an amount of correlation close to 1, which is very high. With this method, similarity values are biased because the average ratings are not very large.

#### 3.2.4. Content-Based Filtering

Content-based filtering (CBF) considers the content of data to recommended relevance based on the user profile to get the user’s attributes of FM and list similar to those liked by the user in the past. Conceptually, this algorithm works by calculating the set of characteristics that are most similar to one given in the user profile, which reflects the long-term past interests, to decide which FM the user will most likely enjoy. In this scenario, the recommender system will learn from the user profile rather than impose upon the user to provide one. The equation describing the process uses restaurant r and user u as input. It uses the degree of user preference the for attribute ri as a weight for the ith component of the distance. The difference between each user–restaurant attribute pair is multiplied by the weight and then summarized. Hence, if there is a perfect match between u and r, the similarity value will be 0. However, if the user has a low score close to 0 for an attribute, it means the user has a negative preference for that specific attribute. In its current form, the next equation does not account for this. A contrary favorite should have the same impact on the final similarity score as a definite preference; below, the Algorithm 2 explains similarities between menus [26]. The hybrid learning method approach will switch between filtering to provide beneficial and accurate suggestion to users after incorporating, and the switch between filtering methods the final rating formula in hybrid learning methods looks like this.


**Algorithm 2: Prediction of hybrid learning incorporating U, FM, and dynamic context**

# switch between U and FM filtering methodsdefine get_hybrid_recs (U_vector, FM)results = {}hybrid_recs $\mathrm{sim}\left(U,\mathrm{FM}\right)=\sqrt{\frac{\sum_{i=1}^{n}{\left(\mathrm{ui}-\mathrm{fmi}\right)}^{2}\times \mathrm{ui}}{\sum_{i=1}^{n}\mathrm{ui}}}$
**# for each FM, calculate the similarity between user and FM**
For FM menu in well-being:result [fm] = simi (user_vector, fm)results. sort ()return resultsdefine get_contrec (matrix, user, location, time):
results = {}for contrec in matrix:
**# identify user context information**
dul == userloca:continuedul = loca value (get-Geographical-MobSetting)
continuedut == currTim:dut = tim value (get-currTime-MobSetting)
**# recommender perf FM list adduser context dynamic information**
Sort list (prefmatrix FM list):return recommender FM list


To get the similarity between U and FM with the highest similarity value that will be recommended to the user, we present several models that can be used to calculate it, and for this system, we decided to use a weighted Euclidean distance measure. This is because we want to be able to assign a level of importance that varies with each attribute, and we would not be able to do this, for example, with a cosine-based measure. The dynamic context information considering the user situation identify pertains to the user’s current geographic location and the time zone setting on the user’s mobile device. This allows the filtering process to generate more similar users having a more significant influence on the final recommendations compared to fewer similar users. The pseudocode explains the recommender FM list using user context with the highest total score that is recommended to the user. The algorithm is effective for the rating production matrix, consequently it is used to normalize these weight ratings.

$$\mathrm{sim}\left(U_{1},U_{2}\right)=\frac{\sum \left(U_{1},U_{2}\right)}{{\sigma U}_{1}\times {\sigma U}_{2}}$$

This proposed algorithm processing requires users to actively participate, as it represents their interests in the profile processing. Based on the learning method’s model, the engine consists of dynamic location information and user profile processing feedback. The personalized inference engine analyzes and processes the content search, while data mash-up, recommendation, and adaptive device integrate with the inference functions. It includes learning methods and the assessment model. Explanations of profiles consist of four attributes, which are restaurant name, location, food menu, and ranking. They are also added as properties for well-being that is food-specific. The content uses content-based filtering in the recommendation algorithms, which can address the cold start problem.

#### 3.2.5. Feedback Loop

The learning method described in Figure 4 is the processing of simulated design based on a feedback loop, which is necessary for understanding user preferences. There are two types of feedback: explicit and implicit. In this paper, we opted to use a rating approach as a means of explicit feedback because reviews can work for sentiment analysis, which is beyond the research scope. The feedback loop design is based on classification knn model processing for 0/1 task, and is deployed in the learning method’s production model. knn classifier is one of the simplest machine learning method algorithms, and the most challenging part is how to determine the value of k when it is too low, as the classifier will be sensitive to noise points, but if it is too big the neighborhood might include too many points from other classes. knn is what we call a lazy learner, meaning that it does not construct a model on which to base future classifications as shown in Algorithm 3 [36]. Every time a new point is classified, it has to calculate k’s nearest neighbors, implying that it does not scale well. The prediction of the learning method’s accuracy overfitting test model is displayed to users, and they have the option of giving feedback. As shown in Table 3, the user can rate the menu on a binary scale, either up or down, and this rating is used to update the user profile.


**Algorithm 3: User Feedback Loop**

define get_Knn_classification (production model)results = {}
**# for each User/FM, full data preparation learning methods using classification knn model processing for (0/1) task**
For start full data preparation:Learning methods [knn, (user_vector, fm)] = similarity (0/1)results. Sort () get accuracy over-fitting testresults recommended optimal (FM).for save and update user profile;return end;return results.


## 4. Experiment and Evaluation

Experiments were conducted to verify the performance of the proposed SRHL. The experiments were carried out by calculating error values that can be derived from incorrect recommendation results when simulated recommendations were performed using the SRHL model. The calculation of error values is computed similarly to the supervised learning [52]. The data for the experiment must already have a value for the recommendation result. When analyzing the actual model, it is assumed that there are no correct answers (recommendation results), and the difference between the finally predicted value and the actual correct answer is defined as an error value. A low error value means that the predicted value is close to the correct answer (the predicted value is highly correlated with the recommendation result that is correct, i.e., similar), and a high error value means that the predicted value is far from correct (the predicted value is less relevant to the recommendation result that is the correct answer). The error values of single models CF and CBF and combinations CF + CBF, CF + ML, and CBF + ML were calculated for comparison with the proposed SRHL, and the experiments proceeded in the following environment, shown in Table 4.

**Figure 6:**
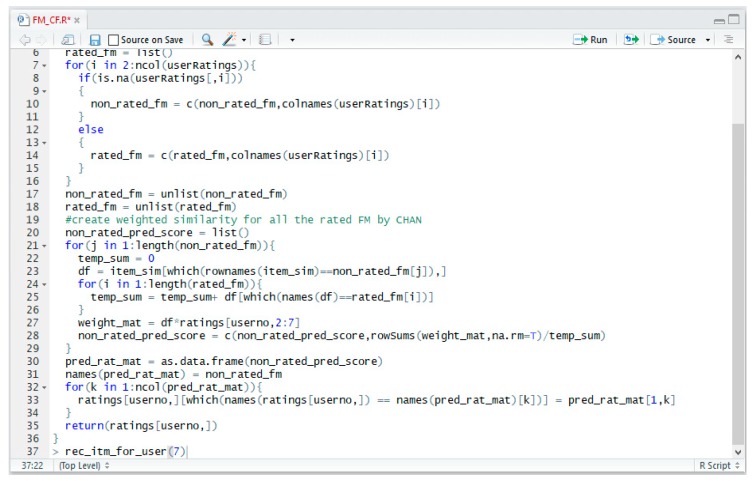
Screenshot of the operation code that is part of recommendation models using R.

**Figure 7:**
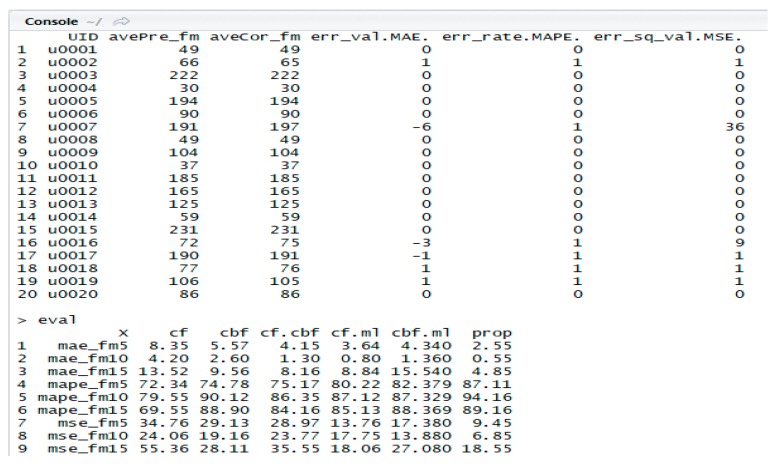
Screenshot of results of MAE, MAPE, and MSE in R.

R language is a powerful tool for data analysis and prediction [53]. R Studio, an integrated development environment, is compatible with various filtering and analysis libraries such as CF, CBF, ML, and similarity [54]. The libraries and functions used in these experiments are shown in Table 5, and a screenshot of the operation code part of the recommendation models is shown in Figure 6 and Figure 7, with the results using three matrix error models (MAE, MAPE, and MSE) for evaluating the recommendation results.

**Figure 8:**
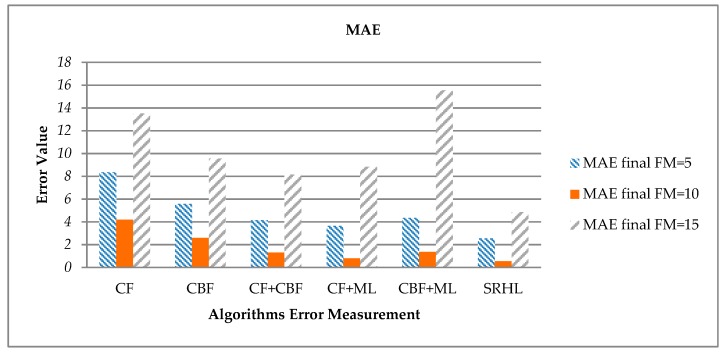
Each experiment’s MAE results.

**Figure 9:**
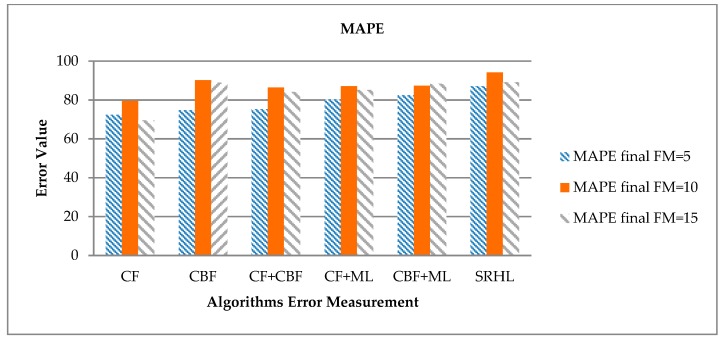
Each experiment’s MAPE results.

**Figure 10:**
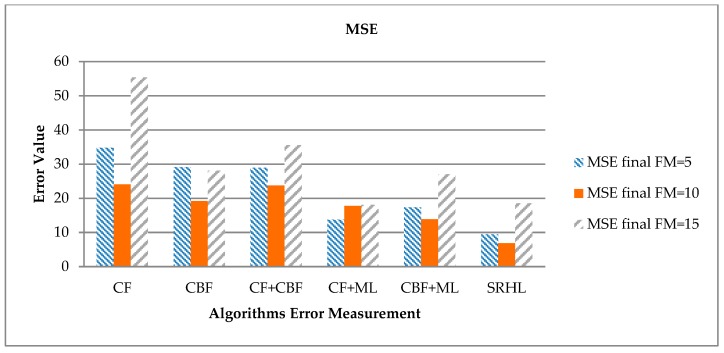
Each experiment’s MSE results.

The code shown in Figure 6 is part of the source code for the CF model of the proposed SRHL, and the console screen in Figure 7 shows the results of MAE, MAPE, and MSE in R. These 20 data points are the result of recommendation results of users and the error values/rates to calculate MAE, MAPE, and MSE when FM = 10. *<avePre_fm>* is a predicted recommendation result, and *<aveCor_fm>* is a correct recommendation result. *<err_val.MAE.>* is a result calculated by the difference between *<avePre_fm>* and *<aveCor_fm>*, which is the error values of users. *<err_rate.MAPE.>* is an error rate for calculating the accuracy through MAPE, which is 1 if there is an error and 0 otherwise. *<err_sq_val.MSE.>* is a squared error value for calculating MSE. In Figure 6, the matrix *<eval>* is derived from six models by calculating the average and frequency of the total results and deriving the final analysis results. The final results are analyzed and summarized in Table 6, Table 7, Table 8, Table 9 and Table 10, and visualized in Figure 8, Figure 9 and Figure 10.

For the experiment we selected close to the FM dominant, this is explicitly and intrinsically, perhaps even directly, related to personal health. The prediction estimates were done among a set of three evaluation criteria matrix error models, explained in Table 6: mean absolute error (MAE), mean absolute percentage error (MAPE), and mean squared error (MSE). The experimental results indicate that the error correlation was compared with the proposed learning methods in SRHL. The experiment used a precollected dataset that included the user profile and history ratings assuming the user feedback after the algorithm was deployed was the same as in the precollected dataset; first FM size sequentially is 5, 10, 15.

FM is a recommendation list that is derived in CF which is the first step in the proposed algorithm for the recommendation. FM decreases in every step of the algorithm because each step deletes 10 useless items. The final numbers are FM-5. It is essential to know the performance of the SRHL after the experiment to find out if the methods have been successful or not. Many properties can be considered when evaluating the recommendation system. The learning methods’ performance can be measured by testing against different datasets using a machine learning method approach and then comparing the performance of the four algorithms (CF + CBF, CF + ML, CBF + ML, CF + CBF + ML) that use these models to calculate accuracy. The first method is used for collaborative filtering (CF) and content-based filtering (CBF), then two algorithms combined (CF + CBF) are applied to CF + ML, then CBF + ML is combined with the final proposed learning method, and then all algorithms (CF + CBF + ML) are combined to forecast the exchange rates. This way, the proposed methods of SRHL calculate the accuracy and reliability of the performance comparison according to three evaluation criteria matrix error [34].

The principal properties of recommendation system accuracy are at a tradeoff when the system wants to focus on diversity. In the formula that describes the process, $d_{i}$ is the actual rating, ${\hat{d}}_{i}$ is the predicted rating, and *n* is the number of items. Also, the value used in the experiment is the content ID of the FM last selected or purchased by the user, the number of materials used in the test is 232, and the content ID has a value of 1–232. The scope of error calculated by MAE and MSE is shown in Table 7. MAPE is an accurate value calculated as a percentage, so it has a range of 0 to 100%. The results of MAPE in the experiment were recalculated to deduct the accuracy of the model as 100% MAPE.

First, the error values derived using MAE are calculated as shown in Table 8 and Figure 8. MAE is a formal method for calculating the average error value that is derived when the model predictions are wrong. From the results, the error values of the proposed SRHL are shown to be low overall. Especially when FM = 10, it can be seen that the low error value is derived from the overall algorithms. FM is the number of recommendations. That is, since the number of items to be recommended is low, if just one of the items is a wrong recommendation, it means that the overall error value may go up. On the contrary, if FM is high, the number of recommended items increases, and the probability of error also increases. That means the number of recommendations, FM, should be set to an appropriate number, and in the present experiment, the error value was low when FM had a median value of about 10.

According to the calculated error when FM = 10, see Table 8 the proposed SRHL showed an error value as low as 3.65 compared to CF. This reduction is the most when compared to all models, because CF shows low recommendation accuracy because of its critical cold start problem. CF + ML showed lower error value than the other models, but the error value was as high as 0.25 compared to the proposed SRHL.

Next, the accuracy calculation results of recommendation using MAPE are shown in Table 9 and Figure 9. MAPE uses the error computed by MAE to convert to a percentage, which is used to indicate the ratio between the zero error value and the others. In this experiment, 100% MAPE is calculated and normalized to express the accuracy of the recommendation rather than the frequency of error that is originally calculated by MAPE. As with MAE, when FM = 10, each model showed the highest recommendation accuracy.

MAPE also showed the lowest accuracy of 79.55% for CF and the proposed SRHL showed the highest recommendation accuracy of 94.16%, which is 14.61% higher than CF. The next highest accuracy for SRHL was 90.12% for CBF. This result was calculated differently from the result of MAE. That means the actual error value of CBF is highly measured, but the percentage of accurate recommendations is high. In other words, CBF is less likely to make a false recommendation, but if it is incorrect, it is accompanied by high error values.

Finally, the calculation results of error numbers using MSE are shown in Table 10 and Figure 10. MSE is a way to calculate error values like MAE. Unlike MAE, it performs a squaring operation on error values to weight against high error values. That is, higher error values are reflected more and lower error values are reflected less.

According to the results, the lowest error value was observed when FM = 10, which showed good results as with MAE. The proposed SRHL showed the lowest error value of 6.85 and the highest error value of 24.06 in CF as well as MAE and MAPE. The proposed SRHL improved the error value by an average of 12.87 over all models when FM = 10. The error value was improved by 17.21 compared to CF, with the highest error value of 24.06, and by 7.03 from 13.88 for CBF+ML, which is the next lowest error value for the proposed SRHL.

According to the analysis carried out above, the best performance was obtained when FM = 10, and a summary of the experimental results is shown in Table 11.

Finally, the three-criteria evaluation matrix, MAE, MAPE, and MSE, were used to measure the performance of the proposed hybrid learning algorithms. The prediction accuracy result of MAE compared to SRHL showed an improvement in performance at approximately 0.55. Furthermore, with MAE the results show a lower value, which is validated as the better result. However, the MAPE result shows an approximately 94.16% higher value, which confirms the better result, while the MSE model’s increase of about 6.85 in value of accuracy over time has become low compared with MAPE, which is stable and the best average model for improved prediction and performance. The proposed SRHL can complement the cold-start problem of CF and the fact that it does not reflect the user’s preference and dynamic profile, which is a disadvantage of CBF, and can recommend items by learning the user’s real-time change through ML. The experimental results show that the performance of the proposed SRHL is better than that of the two single models, CF and CBF, and combinations of the models, CF + CBF, CF + ML, and CBF + ML.

## 5. Conclusions

This paper presents a recommender system using hybrid methods of machine learning for services utilized for personal health and well-being. The recommender used in this method is named SRHL. The crossed learning methods consist of three main straining formulas to improve the restrictions of the slow start with the help of cooperative straining based on the content and the algorithm of the dynamic location in order to clearly specify the evaluation of the user profile. This learning technique enhances the precision of the feedback loop operation formula by knn categorization, which is assigned when k is equal to 1. In that case, the recommender will give suggestions assigned to only the closest neighbor. In order to analyze the effectiveness of the proposed SRHL, the number of materials used in the prediction is estimated, in this case 232. The ID of the content has numerical values of 1 to 232. The reach of errors in accuracy is evaluated by MSE, MAE, and MAPE. The outcomes of the research show that the correlation of error compared with the SRHL method was less than other models. The proposed SRHL showed an error value as low as 3.65 compared to CF, The outcomes indicated that the preciseness will be steady in a complete performance enhancement level, with an efficiency of around 14.61% accuracy, which can be considered low compared to an organizational level. A detailed analysis of the experimental results shows that the SRHL model performed best among all the models such as CF and CBF. The review loop straining process is supposed to enhance the learning techniques to adopt the optimized production and preciseness over the other fitting test. In the system analyzed in this paper, a matrix is used to represent the learning techniques in order to measure and evaluate the precision of identifying services that are optimal for health and lifestyle. The outcomes also indicate that the system can be used to enhance user contentment.

As far as recommendations for future research in the field, other learning methods can also be used to enhance the performance characteristics based on the detailed learning technique formula. This can ensure improved food service suggestions. In addition, hybrid methods can utilize neurobiology concepts on the basis of deep learning methods to offer more evaluation. This can surpass the power to evaluate the hidden factors; for example, the cold start issue to anticipate the commonness in the interaction from the characteristics that could implement enhanced customized RS to improve the experience of a user in a given field.

## Figures and Tables

**Table 1:** Parameters.

Parameters	Description
U	User profile in system database using user-menu rating matrix
	U_n_ number of users in rating matrix
FM	Input number of food menus to be recommended
	FM_n_ number of food menus in rating matrix
K	Number of neighbors used for ranking
Food ID	Dataset of food ID 1–232 used in the test running the models
Sim (U,U_n_)	Euclidean distance to calculate the similarity between two users; it can range from 0 to 1, where 1 represents perfect results of similar Us
si = False	Similarity assuming U has no rating and feedback
si = True	Similarity assuming U has rating and feedback
squares	Add the squares of all differences
prefmatrix	Preferences contain ranks from −1 to 0 to 1 in user-menu rating matrix.

**Table 2:** Menu rating matrix.

	U_1_	U_2_	U_3_	U_4_	U_5_	U_6_	U_7_	U_n_
FM_1_	1	1		1		1	1	
FM_2_	1			1	1	−1	1	
FM_3_	1	1		1		1		
FM_4_		1		1	−1	−1	1	
FM_n_	−1	−1	1		−1	−1	1	

**Table 3:** User preference value changes.

Rating Food Menu (FM) Preference Value Changes
Increased (+v/−v)
Decreased (−v/+v)
FM = [1 0 1 0] U = [0.4 0.6 0.5]
U = [0.4 −v 0.6 + v 0.5 + v]

**Table 4:** Experimental environment.

Element	Performance
OS	Windows 7 Enterprise K Service Pack 1
CPU	Intel(R) Core(TM) i5-3470 CPU @ 3.20 GHz
Memory	8.00 GB
HDD	128 GB SSD
Analysis tool	R-3.5.1 and R studio
Dataset	ID value of 1~232 Source: USDA Branded Food Products Database Software v.3.9.5.1 (released July 2018)

**Table 5:** R library for analysis and prediction of experiments.

Analysis Method	R Library	Function Example
Similarity	proxy	dist (x, method = “cosine”)
	philentropy	distance (x, method = ”euclidean”)
CF	recommenderlab	recommender (x, “UBCF”) predict (x, y, type = “ratings”)
CBF		
ML	caret	train (x, data, method = “*knn*,” metric)
MAE, MAPE, MSE	Metrics	mae (actual, predicted), mse (actual, predicted)

**Table 6:** Formulas of MAE, MAPE, and MSE evaluation criteria.

Evaluation Criterion	Formula	Functional
MAE	$\frac{1}{n}\overset{n}{\underset{1}{\sum }}\left|d_{i}-{\hat{d}}_{i}\right|$	Mean absolute error gives less weight to outliers, as it is not sensitive to outliers.
MAPE	$\frac{100}{n}\overset{n}{\underset{1}{\sum }}\frac{d_{i}-{\hat{d}}_{i}}{d_{i}}$	Mean absolute percentage error is similar to MAE, but is normalized by true observation. The downside is that when true observation is zero, this metric will be problematic.
MSE	$\frac{1}{n}\overset{n}{\underset{1}{\sum }}{\left(d_{i}-{\hat{d}}_{i}\right)}^{2}$	Mean squared error is like a combination measurement of bias and variance of prediction.

**Table 7:** Scope of error that can be calculated by MAE and MSE, and accuracy by MAPE.

Evaluation Criterion	Scope of Results	Description
MAE	0–231	Lower value is better
100% MAPE	0%–100%	Higher value is better
MSE	0–53,361	Lower value is better

**Table 8:** Summary of average error value by MAE.

Model	Model’s Average Error Value Depending on MAE						Proposed Method
**MAE**	**FM**	**CF**	**CBF**	**CF + CBF**	**CF + ML**	**CBF + ML**	**SRHL**
	final FM = 5	8.35	5.57	4.15	3.64	4.34	2.55
	final FM = 10	4.2	2.6	1.3	0.8	1.36	0.55
	final FM = 15	13.52	9.56	8.16	8.84	15.54	4.85

**Table 9:** Recommendation accuracy by MAPE.

Model	Model’s Accuracy Depending on MAPE						Proposed Method
**MAPE**	**FM**	**CF**	**CBF**	**CF + CBF**	**CF + ML**	**CBF + ML**	**SRHL**
	final FM = 5	72.34	74.78	75.17	80.22	82.379	87.11
	final FM = 10	79.55	90.12	86.35	87.12	87.329	94.16
	final FM = 15	69.55	88.9	84.16	85.13	88.369	89.16

**Table 10:** Summary of average error value by MSE.

Model	Model’s Average Error Value Depending on MSE						Proposed Method
**MSE**	**FM**	**CF**	**CBF**	**CF + CBF**	**CF + ML**	**CBF + ML**	**SRHL**
	final FM = 5	34.76	29.13	28.97	13.76	17.38	9.45
	final FM = 10	24.06	19.16	23.77	17.75	13.88	6.85
	final FM = 15	55.36	28.11	35.55	18.06	27.08	18.55

**Table 11:** Result comparison with SRHL and others with FM = 10.

Model	CF	CBF	CF + CBF	CF + ML	CBF + ML	SRHL
MAE	4.2	2.6	1.3	0.8	1.36	0.55
MAPE (%)	79.55	90.12	86.35	87.12	87.329	94.16
MSE	24.06	19.16	23.77	17.75	13.88	6.85
